# Assessing the Degree of Gastroesophageal Reflux Disease (GERD) Knowledge Among the Riyadh Population

**DOI:** 10.7759/cureus.19569

**Published:** 2021-11-14

**Authors:** Omalkhaire M Alshaikh, Issa M Alkhonain, Muath S Anazi, Albaraa A Alahmari, Feras O Alsulami, Abdulrhman A Alsharqi

**Affiliations:** 1 Internal Medicine and Endocrinology, Imam Mohammad Ibn Saud Islamic University, College of Medicine, Riyadh, SAU; 2 Family Medicine, Imam Mohammad Ibn Saud Islamic University, College of Medicine, Riyadh, SAU; 3 Internal Medicine, Imam Mohammad Ibn Saud Islamic University, College of Medicine, Riyadh, SAU; 4 Medicine, Imam Mohammad Ibn Saud Islamic University, College of Medicine, Riyadh, SAU; 5 Emergency Medicine, Al Quwayiyah General Hospital, Riyadh, SAU

**Keywords:** education programme, saudi arabia, prevalence of gerd, riyadh population, disease knowledge, gerd

## Abstract

Background

Gastroesophageal reflux disease (GERD) is a chronic disease mainly characterized by heartburn and acid regurgitation. To our knowledge, there have been a limited number of studies in Saudi Arabia looking at the knowledge level among the general population regarding this disease and its associated factors. Therefore, this study aims to identify the knowledge level of the disease and its associated factors, assess the prevalence of GERD among the Riyadh general population, and assess the need for educational programs for GERD.

Methodology

A cross-sectional study was conducted among the general public in Riyadh, Saudi Arabia. The degree of GERD knowledge was assessed by translating and editing Jorgen Urnes’ 24-item questionnaire into Arabic. In addition, six questions related to the symptoms and complications of GERD were added. Convenience sampling was done by using a Google form to distribute the questionnaire. The questionnaire assesses GERD knowledge by asking about the signs, symptoms, risk factors, predisposing factors, and management of GERD. Statistical analysis was performed using R v. 3.6.3 (https://cran.r-project.org/bin/windows/base/old/3.6.3/). Counts and percentages were used to summarize the distribution of categorical variables.

Results

The questionnaire was completed by 664 respondents (48.2% males and 51.8% females). The average age of the included respondents was 34.1 ± 12.8 years and Saudis represented 97% of the included respondents. The majority of the respondents had heard of GERD (83%). The average number of correct answers was 12.7 ± 6.1. In total, 40 respondents did not answer any questions correctly. Approximately one-third of respondents answered >50% of the questions correctly (n = 250, 37.6%). Approximately half of the respondents identified all risk factors for GERD. Other common risk factors identified included caffeine (23.6%), fast food (26.8%), and smoking (17.6%). Slightly more than a quarter of the respondents reported being diagnosed with GERD (28.8%). Knowledge was significantly higher among respondents who had received a diagnosis of GERD. A statistically significant positive association was observed between age and knowledge (r = 0.19, p < 0.001).

Conclusion

The study shows a relatively good knowledge level compared to previously reported figures in Saudi Arabia and worldwide. Educational programs for GERD should be increased in Saudi Arabia and more health conferences and teaching school students of the disease should be highlighted to increase the general knowledge of this disease in the Kingdom of Saudi Arabia (KSA).

## Introduction

Gastroesophageal reflux disease (GERD) is a chronic disease mainly characterized by heartburn and acid regurgitation [1]. A few patients with reflux symptoms seek healthcare and consult a physician, probably 20-30% [2-3]. Those who ask tend to have more numerous and severe symptoms than those who do not [4]. In the US, many studies have compared the prevalence of GERD among all ethnic groups; they found lower rates of symptoms were reported among Hispanic, Asian, and North American populations [5-6]. A study of an urban population found that 90.3% of diagnosed participants were familiar with GERD, and the information was more frequently gained from the media; on the other hand, there were minimal knowledge and familiarity with different symptoms and complications [6]. Patient education exerts its effect through patient "learning."

Only a few studies have evaluated the impact of patient education and the relationship between patient learning and quality of life [7-8], In some chronic diseases, patient education has been shown to decrease symptoms and number of visits to the physician and reduce health-related costs [9-11]. A Norwegian study about patient education was designed as a structured dialog transmitting medical information about the pathophysiology and prognosis, the pharmacological and nonpharmacological treatment of GERD, patients’ rights, and the use of healthcare. Outcomes were measured using general QoL [General Health Questionnaire-30 (GHQ-30)] and disease-specific QoL [Digestive Symptoms and Impact Questionnaire (DSIQ)]. They found that patients with primary school education only, minor psychiatric disease, and a low QoL achieved a lower knowledge level and deserved special attention in future educational programs. Changes in knowledge were not associated with changes in quality of life, which indicates a complex association between the two [12-13]. A study in the US about the prevalence and awareness of and care patterns for GERD among minority populations designed a questionnaire with 12 domains: (1) familiarity with the term GERD; (2) incidence of heartburn; (3) diagnosis of GERD; (4) medication use; (5) social impact; (6) attitudes about seeking care; (7) knowledge about heartburn or GERD; (8) sources of information; (9) beliefs about symptomatic relief for heartburn; (10) beliefs about symptoms associated with heartburn; (11) intentions for treating heartburn; (12) demographics (including gender, marital status, education, employment status, income, race. ethnicity, weight, and height). They found the following prevalence rates between racial groups: 50% of Hispanics experienced heartburn at least monthly compared with 37% of Caucasians, 31% of African-Americans, and 20% of Asians. Significant differences in knowledge and care-seeking patterns by ethnicity were also observed; 74.4% of those with GERD symptoms were familiar with GERD. Although Hispanics had the highest prevalence rate for GERD, their familiarity with GERD was lower (72.4%) than that of Caucasians (78.2%), Asians (75%), and African-Americans (72.7%) [14].

The prevalence of GERD deferent depends on ethnicity [14]; it is about 10-20% in western countries [15-17] and 2.5-8.5% in Asian countries [18-20]. Patient education in some chronic diseases shows benefits [21-25], and the GERD education program shows improvement in patient quality of life [12].

The GERD study group of the Korean Society of Neurogastroenterology and Motility studied the degree of knowledge among 746 Korean patients with GERD regarding their disease. The patients’ ages were 15-91 years and the prospective survey included patients diagnosed with GERD who were able to complete the survey; it was published in July 2017 [26]. They assessed the degree of disease knowledge by translating Urnes’ descriptive survey items into Korean [13]. The survey answers were “true,” “false,” or “don’t know” and the score range was 0-22. The questions were about some symptoms, relations to certain foods, and they asked about the investigations, treatment, prognosis, and complications. In addition, they asked about the patients’ sociodemographic characteristics, what the patients wanted to know about GERD, the first time the patients heard about GERD, and what they consider the best source of information about GERD. The result shows that the mean percentage of correct answers was 46.3% and the mean GERD knowledge score was 9.6 [26]. The findings reported in Urnes showed a mean GERD knowledge score of 13.1 [13]. The limitation of this study is that they used a questionnaire that was validated after translation, which might lead to bias due to differences in language and culture. They suggested that a larger study with a self-developed questionnaire may obtain more accurate information [26].

In January 2018, a published study assessed the knowledge of GERD in Al-Taif, Saudi Arabia [27]. the study aimed to assess the knowledge of signs, symptoms, and risk factors of GERD among the Saudi population in Al-Taif. They administered a questionnaire to a nonrandom purposive sample of 202 Saudi participants who lived in Al-Taif. The questionnaire was adapted from minimal medical knowledge (MMK), and they focused on basic questions about common signs, symptoms, and risk factors of GERD. The questions could be answered as yes or no, and the questionnaire was split into two parts: the first asked about sociodemographic characteristics and the second asked about the participant’s knowledge of GERD. The mean MMK score was 64.66%; one-third of participants had either personal experience of GERD or a family history of GERD. In addition, they found that the higher the level of education, the higher the score, and the participants had gained most of their knowledge from the internet and books. They recommended providing health information about GERD on the internet or preparing handouts and distributing them through health care facilities, and they mentioned that there has been no study in Saudi Arabia that assesses the knowledge level of GERD. The limitations of the study were that most of their participants had a higher education level and one-third of participants had a medical background [27].

Elnemr and Almuntashiri performed a cross-sectional observational study to predict the prevalence of gastroesophageal reflux disease (GERD) and risk factors related to GERD among male students at Taif University [28]. Elnemr and Almuntashiri found that more than half of the 464 male students surveyed at Taif University (53.2%) suffered from GERD. Their study demonstrated that rates of obesity, drinking soft drinks often, and stress levels all have a stronger influence on GERD disease. In addition, there was a significant association between smoking and GERD with a higher prevalence (68.3%) than among non-smokers. Elnemr and Almuntashiri demonstrated that there was a significant association between past medical history elements, such as high blood pressure (84.2%), psychiatric diseases (75.0%), diabetes mellitus (66.7%), irritable bowel syndrome (66.7%), and asthma (50%), with GERD [28].

A cohort study of the prevalence of symptoms of gastroesophageal reflux disease (GERD) done in the Riyadh region by Almadi [29] showed that 45.4% of the 1,265 total participants suffer from GERD based on the Gastroesophageal Reflux Disease Questionnaire (GerdQ). Sixty-seven point eighty-one percent of the sample were female and 62.73% of them suffered from GERD once or more a week. Meanwhile, there were no variations in GERD prevalence between the genders with males (45.43%) and females (45.13%). The study illustrated that older age, higher body mass index (BMI), and smokers tended to have a higher prevalence of GERD [29].

Regarding the prevalence and risk factors of gastroesophageal reflux diseases among Shaqra University students, According to American Nutrition Association, 70 million people suffer from digestive issues daily [30] and GERD is one of the most common GI diseases, affecting millions worldwide [31]. The study aimed to measure the prevalence of GERD and determine its risk factors among undergraduate students at Shaqra University. Several risk factors are associated with GERD, including social habits such as smoking, physical activity, high BMI, and high salt and fast food consumption [5,32]. The researchers conducted a cross-sectional study using a structured questionnaire distributed among Shaqra University students through a multistage stratification and random sampling technique to divide students in terms of gender and the three colleges at the university. The study intended to use and assess the questionnaire to measure the prevalence of GERD and its risk factors. The baseline sample size was 435, of whom 400 were included in the study [227 (56%) male and 173 (43%) female] with ±5% precision and a 95% confidence interval (CI). The study revealed that the prevalence of GERD is 23.8% among students at Shaqra University. The study also showed that male participants had a slightly higher prevalence due to the age group of the participants and the manner of their social life, where most of them lived in a dorm and experienced stress due to exams and isolation. It showed that a high percentage of them developed unhealthy habits such as smoking, continuous fast-food eating, eating quickly, sleeping less than an hour after a meal, and drinking carbonated drinks, all of which are risk factors for developing GERD. A comparison between this study and other studies was performed in Al-Taif, KSA [33]. The results reveal a higher incidence in the samples in this study. Alongside other studies that were conducted in Syria and India [34-35]. This result shows that the prevalence is also higher in Shaqra University, which can be related to the above-mentioned facts regarding living in a dormitory, the age of the participants, and their unhealthy lifestyles.

To our knowledge, few studies in Saudi Arabia have examined the knowledge level of the general population regarding the disease and its associated factors. Therefore, this study aims to identify the knowledge level of the disease and its associated factors, assess the prevalence of GERD among the Saudi general population, and assess the need for educational programs for GERD.

## Materials and methods

A cross-sectional study was conducted among the general public in Riyadh, Saudi Arabia, not entire Saudi Arabia, as it would have an insufficient number of participants in some cities and be time-consuming. The degree of GERD knowledge was assessed by using the Jorgen Urnes [12] questionnaire after taking permission from Jurgen Urnes, the questionnaire was translated and edited to fit the Arabic language and make it clear to participants when they read it.

Convenience sampling was done using a Google form to distribute the questionnaire. The questionnaire assesses GERD knowledge by asking about signs, symptoms, risks factors, predisposing factors, and the management of GERD. We included all Riyadh residents and excluded anyone who works in the health field. Our criteria for inclusion were all Riyadh residents, including both men and women. Our exclusion criterion was everyone who works in healthcare.

Knowledge scoring

The GERD knowledge test originally included 24 questions related to GERD [12]. The patients responded to these statements with either "true," "false," or "don't know." In addition, six questions related to the symptoms and complications of GERD were added. The GERD knowledge test score (GERD-knowledge) was the sum of correct responses with a possible score of 0-30.

Statistical analysis

Statistical analysis was performed using R v. 3.6.3 (https://cran.r-project.org/bin/windows/base/old/3.6.3/). Counts and percentages were used to summarize the distribution of categorical variables. The mean ± standard deviation was used to summarize the distribution of continuous variables. The chi-square test of independence was used to assess the association between categorical variables. Multivariate linear regression was used to assess sociodemographic factors associated with the knowledge score. Pearson’s correlation was used to assess the association between age and knowledge score. Hypothesis testing was performed at the 5% significance level.

## Results

The questionnaire was completed by 664 respondents (48.2% males and 51.8% females). The average age of the included respondents was 34.1 ± 12.8 years, and Saudis represented 97% of the included respondents. Regarding education, more than half of the respondents had a bachelor’s degree (58.1%) and 11.3% had a post-graduate degree. Respondents working in the public sector represented one-third of the study sample, 20.8% were private-sector employees, 20% were students, and 8.89% were retired. The average monthly income was <5,000. Three-quarters of the respondents were prescribed medications (72.4%) for 33.7% of the respondents, 5000-1000 SAR for less than a quarter (23.9%), and 10,001-15,000 SAR for 23%. The remaining 8.89% and 10.4% reported an average monthly income of 15,001-20,000 and >20,000 SAR, respectively (Table 1).

**Table 1:** Descriptive statistics for the study sample

	[ALL]
	N = 664
Gender:	
Female	344 (51.8%)
Male	320 (48.2%)
Age:	34.1 (12.8)
Nationality:	
Non-Saudi	20 (3.01%)
Saudi	644 (97.0%)
Education:	
Primary	3 (0.45%)
Secondary	32 (4.82%)
High school	126 (19.0%)
Diploma	42 (6.33%)
University	386 (58.1%)
Post-graduate	75 (11.3%)
Occupation:	
Government employee	200 (30.1%)
Housewife	41 (6.17%)
Medical	1 (0.15%)
Other	1 (0.15%)
Private sector employee	138 (20.8%)
Retired	59 (8.89%)
Self-employed	3 (0.45%)
Student	132 (19.9%)
Unemployed	89 (13.4%)
Monthly income:	
<5,000 SAR	224 (33.7%)
5,000–10,000 SAR	159 (23.9%)
10,001–15,000 SAR	153 (23.0%)
15,001–20,000 SAR	59 (8.89%)
>20,000 SAR	69 (10.4%)
Data were summarized using mean ± SD for age and counts (%) for the remaining variables.	

The majority of the respondents had heard of GERD (83%). More than half of the respondents (59.2%) thought that herbal medicine was not related to GERD, and about one-quarter thought that it could cause GERD (25.2%). Slightly more than a quarter of the respondents reported being diagnosed with GERD (28.8%). Of these, endoscopies were performed in 48.2% and almost all were prescribed medication for GERD (91.1%). Three-quarters of the respondents who were prescribed medications (72.4%) were compliant (Table 2).

**Table 2:** Diagnosis of GERD in the included respondents GERD: gastroesophageal reflux disease

	[ALL]	N
	N = 664	
Ever heard of GERD:		664
No	113 (17.0%)	
Yes	551 (83.0%)	
Herbal/Folk medicine:		664
Can cause GERD	167 (25.2%)	
Can treat GERD	104 (15.7%)	
Not related to GERD	393 (59.2%)	
Ever diagnosed with GERD:		664
No	473 (71.2%)	
Yes	191 (28.8%)	
Endoscopy at the time of diagnosis:		191
No	99 (51.8%)	
Yes	92 (48.2%)	
Prescribed any medicines for GERD:		191
No	17 (8.90%)	
Yes	174 (91.1%)	
Compliant with the prescribed medications:		174
No	48 (27.6%)	
Yes	126 (72.4%)	
Data were summarized using counts (%).		

**Figure 1:**
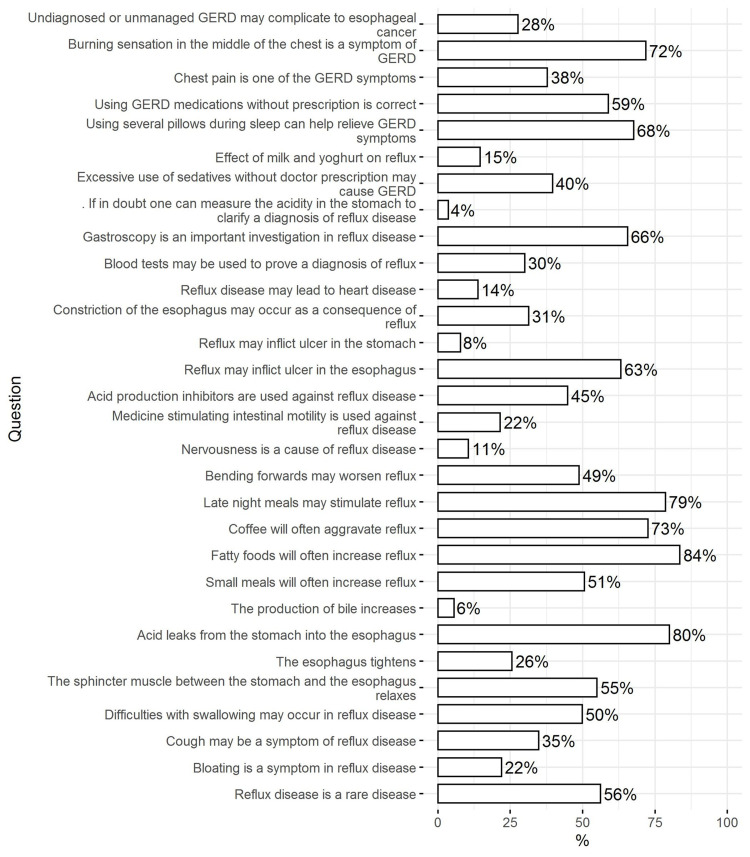
Knowledge regarding GERD stratified by diagnosis status (% of correct answers is shown for each question) GERD: gastroesophageal reflux disease

The results show that the percentage of correct answers varied in the range of 4-84%. Only 4% knew that stomach acidity cannot be used to confirm a diagnosis of GERD and only 6% knew that the production of bile does not increase. Only 8% knew that GERD cannot cause gastric ulcers, and 15% knew that milk and yogurt may aggravate ulcers. The majority of respondents knew that fatty acids can increase reflux (84%) and 80% knew that acid leaks from the stomach into the esophagus. Roughly similar numbers (79% and 73%) knew that light meals cannot stimulate reflux and that coffee may aggravate reflux symptoms, respectively. Only half of the respondents knew that bending down could aggravate reflux. More than half of respondents knew that using medications for GERD without prescription is not correct (59%). Only 14% knew that GERD cannot lead to heart disease (Figure 1).

The results showed that knowledge was significantly higher in respondents diagnosed with GERD. Three-quarters of the respondents diagnosed with GERD knew that it was not a rare disease (73.8%) compared with only 49% of respondents not diagnosed with GERD (p < 0.001). Half of the respondents diagnosed with GERD identified cough as a potential symptom (49.7%) compared to only 28.8% of those not diagnosed with GERD (p < 0.001). More than two-thirds of patients diagnosed with GERD knew that it could be associated with difficulty swallowing compared to 42.9% of respondents not diagnosed with GERD (p < 0.001). Almost all patients diagnosed with GERD knew that acid leaks into the esophagus (94.8%) compared to 74% of respondents who were not diagnosed with GERD (p < 0.001). Knowledge regarding the production of bile, the ulcerogenic effect of GERD on the stomach, and chest pain associated with GERD were low in both groups, although it was higher in patients diagnosed with GERD. Knowledge regarding the effect of GERD on the heart and the usefulness of blood tests were low in both groups and the difference was not statistically significant (p > 0.05). Knowledge regarding the usefulness of acidity testing was very low in both groups and the proportion of respondents who chose the wrong answer was higher among respondents diagnosed with GERD (p < 0.05). Knowledge regarding the harmful effects of sedatives was not significantly different between groups (Table 3).

**Table 3:** Knowledge regarding GERD stratified by diagnosis GERD: gastroesophageal reflux disease

	[ALL]	No	Yes	p
	N = 664	N = 473	N = 191	
Reflux disease is a rare disease:				<0.001
Don't know	255 (38.4%)	212 (44.8%)	43 (22.5%)	
FALSE*	373 (56.2%)	232 (49.0%)	141 (73.8%)	
TRUE	36 (5.42%)	29 (6.13%)	7 (3.66%)	
Bloating is a symptom in reflux disease:				<0.001
Don't know	328 (49.4%)	260 (55.0%)	68 (35.6%)	
FALSE*	146 (22.0%)	90 (19.0%)	56 (29.3%)	
TRUE	190 (28.6%)	123 (26.0%)	67 (35.1%)	
Cough may be a symptom of reflux disease:				<0.001
Don't know	299 (45.0%)	241 (51.0%)	58 (30.4%)	
FALSE	134 (20.2%)	96 (20.3%)	38 (19.9%)	
TRUE*	231 (34.8%)	136 (28.8%)	95 (49.7%)	
Difficulties with swallowing may occur in reflux disease:				<0.001
Don't know	263 (39.6%)	223 (47.1%)	40 (20.9%)	
FALSE	70 (10.5%)	47 (9.94%)	23 (12.0%)	
TRUE*	331 (49.8%)	203 (42.9%)	128 (67.0%)	
The sphincter muscle between the stomach and the esophagus relaxes:				<0.001
Don't know	271 (40.8%)	223 (47.1%)	48 (25.1%)	
FALSE	28 (4.22%)	22 (4.65%)	6 (3.14%)	
TRUE*	365 (55.0%)	228 (48.2%)	137 (71.7%)	
The esophagus tightens:				0.001
Don't know	322 (48.5%)	251 (53.1%)	71 (37.2%)	
FALSE*	170 (25.6%)	107 (22.6%)	63 (33.0%)	
TRUE	172 (25.9%)	115 (24.3%)	57 (29.8%)	
Acid leaks from the stomach into the esophagus:				<0.001
Don't know	128 (19.3%)	118 (24.9%)	10 (5.24%)	
FALSE	5 (0.75%)	5 (1.06%)	0 (0.00%)	
TRUE*	531 (80.0%)	350 (74.0%)	181 (94.8%)	
The production of bile increases:				0.002
Don't know	307 (46.2%)	238 (50.3%)	69 (36.1%)	
FALSE*	37 (5.57%)	27 (5.71%)	10 (5.24%)	
TRUE	320 (48.2%)	208 (44.0%)	112 (58.6%)	
Small meals will often increase reflux:				<0.001
Don't know	205 (30.9%)	175 (37.0%)	30 (15.7%)	
FALSE*	336 (50.6%)	218 (46.1%)	118 (61.8%)	
TRUE	123 (18.5%)	80 (16.9%)	43 (22.5%)	
Fatty foods will often increase reflux:				<0.001
Don't know	94 (14.2%)	84 (17.8%)	10 (5.24%)	
FALSE	15 (2.26%)	11 (2.33%)	4 (2.09%)	
TRUE*	555 (83.6%)	378 (79.9%)	177 (92.7%)	
Coffee will often aggravate reflux:				<0.001
Don't know	146 (22.0%)	129 (27.3%)	17 (8.90%)	
FALSE	36 (5.42%)	28 (5.92%)	8 (4.19%)	
TRUE*	482 (72.6%)	316 (66.8%)	166 (86.9%)	
Late night meals may stimulate reflux:				<0.001
Don't know	123 (18.5%)	109 (23.0%)	14 (7.33%)	
FALSE	19 (2.86%)	14 (2.96%)	5 (2.62%)	
TRUE*	522 (78.6%)	350 (74.0%)	172 (90.1%)	
Bending forwards may worsen reflux:				<0.001
Don't know	269 (40.5%)	216 (45.7%)	53 (27.7%)	
FALSE	71 (10.7%)	54 (11.4%)	17 (8.90%)	
TRUE*	324 (48.8%)	203 (42.9%)	121 (63.4%)	
Nervousness is a cause of reflux disease:				<0.001
Don't know	347 (52.3%)	271 (57.3%)	76 (39.8%)	
FALSE*	70 (10.5%)	57 (12.1%)	13 (6.81%)	
TRUE	247 (37.2%)	145 (30.7%)	102 (53.4%)	
Medicine stimulating intestinal motility is used against reflux disease:				<0.001
Don't know	433 (65.2%)	342 (72.3%)	91 (47.6%)	
FALSE	88 (13.3%)	48 (10.1%)	40 (20.9%)	
TRUE*	143 (21.5%)	83 (17.5%)	60 (31.4%)	
Acid production inhibitors are used against reflux disease:				<0.001
Don't know	349 (52.6%)	300 (63.4%)	49 (25.7%)	
FALSE	17 (2.56%)	14 (2.96%)	3 (1.57%)	
TRUE*	298 (44.9%)	159 (33.6%)	139 (72.8%)	
Reflux may inflict ulcer in the esophagus:				0.002
Don't know	236 (35.5%)	187 (39.5%)	49 (25.7%)	
FALSE	8 (1.20%)	5 (1.06%)	3 (1.57%)	
TRUE*	420 (63.3%)	281 (59.4%)	139 (72.8%)	
Reflux may inflict ulcer in the stomach:				0.069
Don't know	316 (47.6%)	237 (50.1%)	79 (41.4%)	
FALSE*	52 (7.83%)	32 (6.77%)	20 (10.5%)	
TRUE	296 (44.6%)	204 (43.1%)	92 (48.2%)	
Constriction of the esophagus may occur as a consequence				0.004
Don't know	374 (56.3%)	284 (60.0%)	90 (47.1%)	
FALSE	82 (12.3%)	58 (12.3%)	24 (12.6%)	
TRUE*	208 (31.3%)	131 (27.7%)	77 (40.3%)	
Reflux disease may lead to heart disease:				0.930
Don't know	501 (75.5%)	356 (75.3%)	145 (75.9%)	
FALSE*	92 (13.9%)	67 (14.2%)	25 (13.1%)	
TRUE	71 (10.7%)	50 (10.6%)	21 (11.0%)	
Blood tests may be used to prove a diagnosis of reflux:				0.126
Don't know	420 (63.3%)	308 (65.1%)	112 (58.6%)	
FALSE*	199 (30.0%)	131 (27.7%)	68 (35.6%)	
TRUE	45 (6.78%)	34 (7.19%)	11 (5.76%)	
Gastroscopy is an important investigation in reflux disease:				<0.001
Don't know	209 (31.5%)	172 (36.4%)	37 (19.4%)	
FALSE	20 (3.01%)	14 (2.96%)	6 (3.14%)	
TRUE*	435 (65.5%)	287 (60.7%)	148 (77.5%)	
If in doubt one can measure the acidity in the stomach to clarify a diagnosis of reflux disease:				0.005
Don't know	351 (52.9%)	267 (56.4%)	84 (44.0%)	
FALSE*	24 (3.61%)	19 (4.02%)	5 (2.62%)	
TRUE	289 (43.5%)	187 (39.5%)	102 (53.4%)	
Excessive use of sedatives without doctor prescription may cause GERD:				0.501
Don't know	379 (57.1%)	264 (55.8%)	115 (60.2%)	
FALSE	22 (3.31%)	15 (3.17%)	7 (3.66%)	
TRUE*	263 (39.6%)	194 (41.0%)	69 (36.1%)	
Effect of milk and yoghurt on reflux:				<0.001
Aggravate reflux*	97 (14.6%)	52 (11.0%)	45 (23.6%)	
Don't know	225 (33.9%)	188 (39.7%)	37 (19.4%)	
No effect	70 (10.5%)	40 (8.46%)	30 (15.7%)	
Relieve reflux	272 (41.0%)	193 (40.8%)	79 (41.4%)	
Using several pillows during sleep can help relieve GERD symptoms:				<0.001
Don't know	186 (28.0%)	161 (34.0%)	25 (13.1%)	
FALSE	29 (4.37%)	21 (4.44%)	8 (4.19%)	
TRUE*	449 (67.6%)	291 (61.5%)	158 (82.7%)	
Using GERD medications without prescription is correct:				<0.001
Don't know	205 (30.9%)	170 (35.9%)	35 (18.3%)	
FALSE*	391 (58.9%)	267 (56.4%)	124 (64.9%)	
TRUE	68 (10.2%)	36 (7.61%)	32 (16.8%)	
Chest pain is one of the GERD symptoms:				<0.001
Don't know	320 (48.2%)	267 (56.4%)	53 (27.7%)	
FALSE*	93 (14.0%)	69 (14.6%)	24 (12.6%)	
TRUE	251 (37.8%)	137 (29.0%)	114 (59.7%)	
Burning sensation in the middle of the chest is a symptom				<0.001
Don't know	165 (24.8%)	156 (33.0%)	9 (4.71%)	
FALSE*	22 (3.31%)	17 (3.59%)	5 (2.62%)	
TRUE	477 (71.8%)	300 (63.4%)	177 (92.7%)	
Undiagnosed or unmanaged GERD may complicate to esophageal cancer:				0.011
Don't know	454 (68.4%)	332 (70.2%)	122 (63.9%)	
FALSE	26 (3.92%)	23 (4.86%)	3 (1.57%)	
TRUE*	184 (27.7%)	118 (24.9%)	66 (34.6%)	
Counts and percentages were used to summarize responses The chi-squared test of independence was used to assess the association between variables				

**Figure 2:**
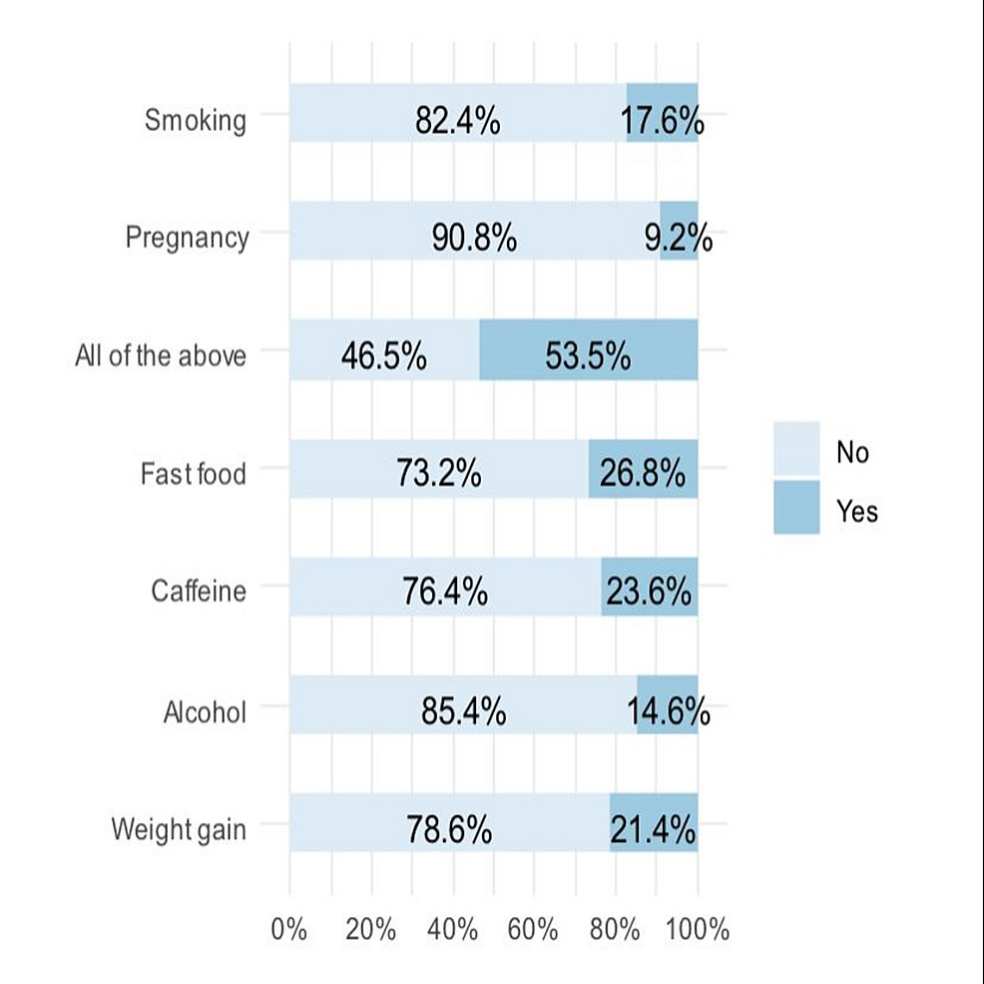
Risk factors for GERD identified by the respondents GERD: gastroesophageal reflux disease

Approximately half of the respondents identified all risk factors for GERD. Other common risk factors identified included caffeine (23.6%), fast food (26.8%), and smoking (17.6%) (Figure 2).

**Figure 3:**
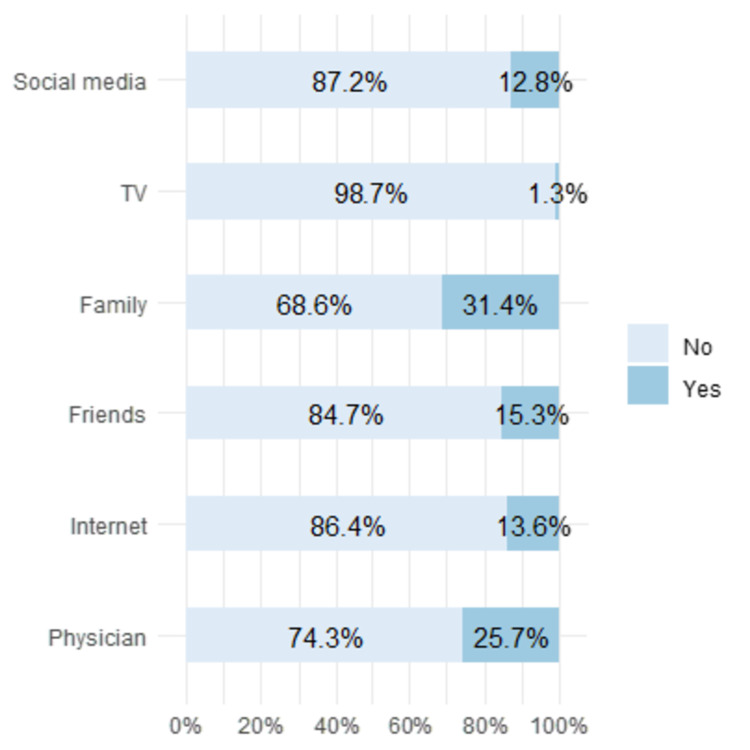
Sources of information regarding GERD GERD: gastroesophageal reflux disease

Of the 577 respondents who had heard of GERD, the most common sources of information regarding GERD were family (31.4%), physicians (25.7%), friends (15.3%), and social media (12.8%) (Figure 3).

**Figure 4:**
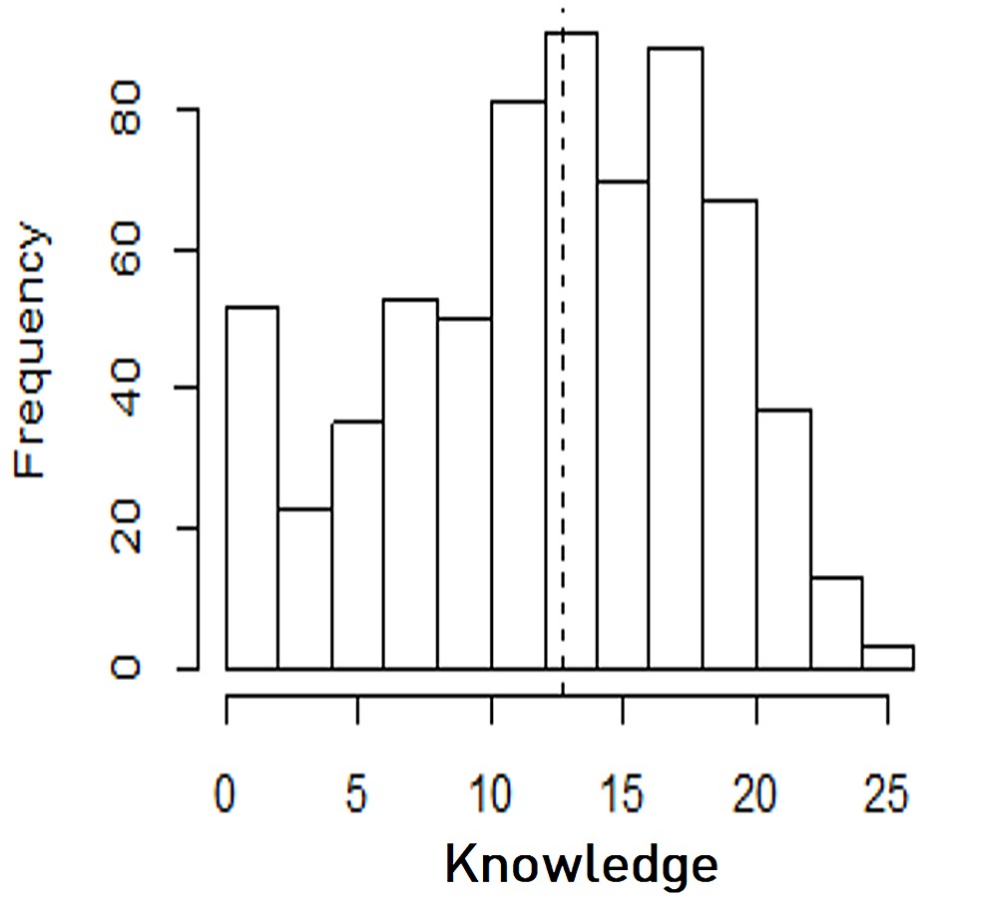
Distribution of correct answers

The average number of correct answers was 12.7 ± 6.1. A total of 40 respondents did not answer any question correctly and approximately one-third of the respondents answered >50% of the questions correctly (n = 250, 37.6%) (Figure 4). Sixteen respondents answered >22 questions correctly and 53 completed more than two-thirds of the questions correctly. Linear regression was used to assess the factors associated with knowledge. Independent variables included age, gender, nationality, occupation, monthly income, and GERD diagnosis (Table 4).

**Table 4:** Factors associated with knowledge score GERD: gastroesophageal reflux disease

Predictors	Estimates	CI	p
(Intercept)	4.64	1.21–8.07	0.008
Age	0.06	0.00–0.11	0.035
Female	Reference		
Male	-1.29	-2.23– -0.35	0.007
Non-Saudi	Reference		
Saudi	0.98	-1.45–3.41	0.429
Education: University	Reference		
Education: High school	-1.08	-2.21–0.06	0.063
Education: Diploma	-0.24	-1.94–1.46	0.782
Education: Post-graduate	-0.62	-1.99–0.74	0.373
Heard of GERD: No	Reference		
Heard of GERD: Yes	5.39	4.23–6.55	<0.001
Income category	0.08	-0.31–0.47	0.689
Occupation: Government employee	Reference		
Occupation: Other	-1.50	-3.42–0.42	0.125
Occupation: Private-sector employee	-0.04	-1.23–1.15	0.948
Occupation: Retired	1.22	-0.62–3.07	0.194
Occupation: Student	2.45	0.83–4.07	0.003
Occupation: Unemployed	1.06	-0.43–2.55	0.161
Diagnosed with GERD: No	Reference		
Diagnosed with GERD: Yes	3.09	2.14–4.03	<0.001

Results showed that multiple factors were associated with knowledge regarding GERD. Higher age was associated with more knowledge about GERD (B = 0.06, p = 0.035). Knowledge was significantly lower among men than women (B = -1.29, p = 0.007). Nationality was not associated with knowledge regarding GERD. Respondents with a high-school education level had a significantly lower knowledge score than respondents with a university education (B = -1.08, p = 0.063) although the association was statistically significant at the 0.1 level. Knowing about GERD was associated with better knowledge (B = 5.39, p < 0.001), and a diagnosis of GERD was also associated with a higher average knowledge score (B = 3.09, p < 0.001). The average knowledge score was significantly higher among students than among respondents working as government employees (B = 2.45, p = 0.003).

**Figure 5:**
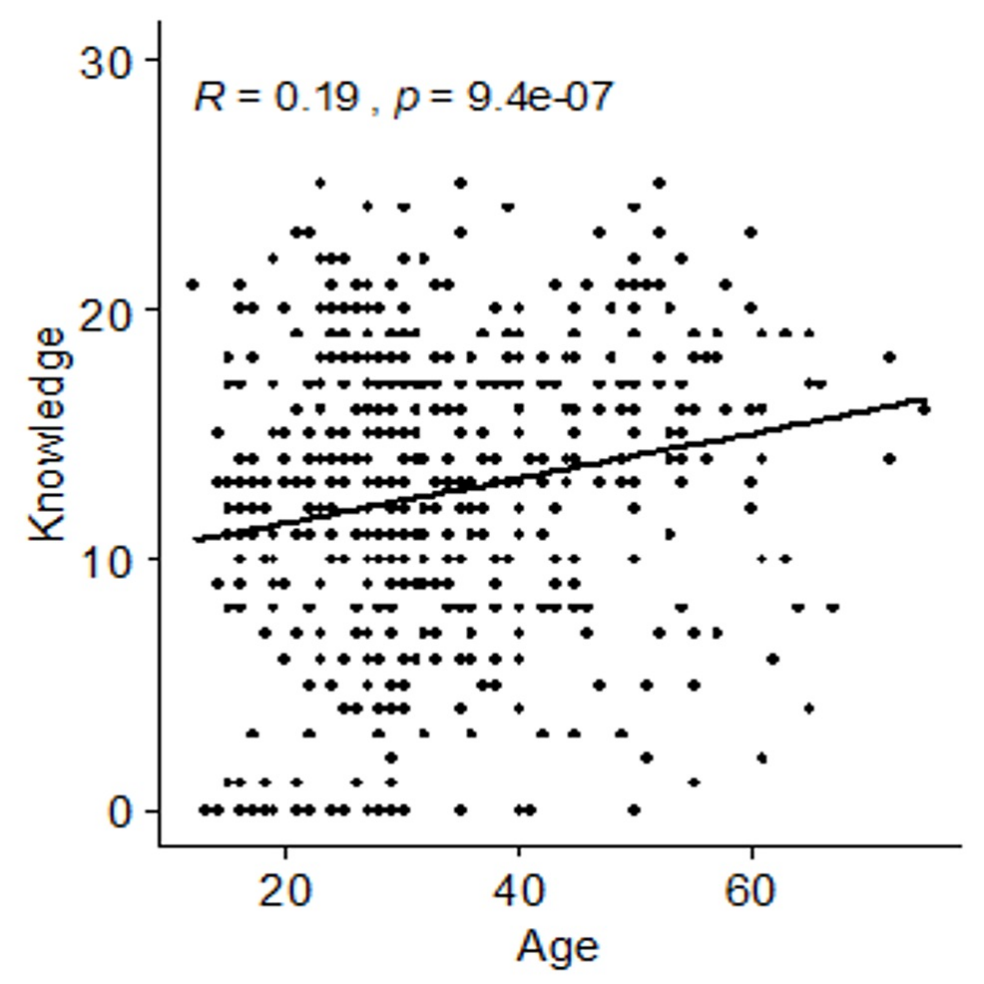
Association between age and knowledge score

A statistically significant positive association was observed between age and knowledge (r = 0.19, p < 0.001) (Figure 5).

## Discussion

GERD is a widespread disorder that places a considerable financial strain on the healthcare system owing to management expenditures and significantly impacts afflicted people’s quality of life. The prevalence of GERD has increased in recent decades, and it is now one of the most common chronic diseases [6]. The actual benefits of this study for participants are increasing knowledge about and the level of awareness regarding GERD and its complications among the Riyadh population and comparing between different groups in the target population, such as different genders, education levels, monthly income, GERD patients, and others, to see the difference between their awareness of this disease and its complications. In addition, the actual benefits for society include driving the efforts of the local health education programs toward real needs regarding GERD and trying to focus on specific groups in a population if a disparity exists in the level of awareness.

The prevalence of GERD was assessed in different regions in Saudi Arabia and other Arabian countries. According to our study results, about a quarter of respondents reported being diagnosed with GERD (28.8%). Of these, endoscopy was performed in 48.2% and almost all were prescribed medications for GERD (91.1%). Three-quarters of the respondents who were prescribed medications (72.4%) were compliant. This was much lower than reported in a Saudi study in the Arar region, as GERD was found to be prevalent in 61.8% of the study participants. Among this prevalence of GERD, there was no significant influence of sex, age, educational level, marital status, or employment status [36]. In Al-Taif, one-third of participants had personal experience of GERD [27]. Another study in Taif among university students reported a prevalence of GERD (at 53.2%) among students [28]. In the Riyadh region, a cohort study of the prevalence of symptoms of GERD showed that 45.4% of the 1,265 participants in total suffer from GERD based on GerdQ [29].

In our study, the average score of correct answers was 12.7 ± 6.1. This was lower than the results reported by Mohammad. In a different region of Saudi Arabia where the average degree of awareness was discovered to be 20.8%. Most of the participants (341 or 74.3% of the total) had an excellent understanding of GERD [37]. A Korean study showed the mean percentage of correct answers was 46.3% and the mean GERD knowledge score was 9.6 [26]. Another study finding reported a mean GERD knowledge score of 13.1 [13]. In Al-Taif, KSA, the mean MMK score was 64.66% [27].

The results of our study showed that knowledge was significantly higher among respondents diagnosed with GERD. GERD education must be delivered during doctor visits and in communities. Soliciting information on classic and accompanying symptoms will enhance patient awareness regarding symptoms that they may ascribe to indigestion or minor diseases. A complete medication history of all prescribed, over-the-counter, and alternative/home treatments may also give a window of opportunity to diagnose and intervene on concurrent GERD-like symptoms. Using a validated culturally sensitive questionnaire with words and images will help improve communication about symptoms and therapy [38]. In one previous study, a GERD educational program was useful for increasing disease knowledge but did not improve the quality of life for all patients [12].

Excessive junk food consumption, increasing consumption of soda and other high sugar goods, lack of physical activity, excessive and extended smoking, and drunkenness are all risk factors for GERD. Along with lifestyle, additional variables, such as genetic predisposition and rising age (older than 50 years), are linked to an increased risk of GERD. Furthermore, due to the additional pressure on the stomach and other organs caused by a fetus, pregnant women are at a higher risk of having transient GERD. Furthermore, therapeutic medications used to treat certain connective diseases and pharmaceuticals, such as analgesics, antidepressants, anticholinergic agents, calcium channel blocking agents, and blood sugar-regulating drugs, may raise the risk of GERD in some people [2]. Approximately half of the respondents in our study identified all risk factors for GERD. Other common risk factors identified included caffeine (23.6%), fast food (26.8%), and smoking (17.6%). More than half of respondents (59.2%) thought that herbal medicine was not related to GERD while about one-quarter thought that it can cause GERD (25.2%). This was comparable to the results of previous studies as in Korea, where the degree of knowledge (mean percentage of correct answers) regarding the etiology, prognosis, and treatment of GERD were 49.5%, 36.7%, and 37.5%, respectively [26]. Du Jeong also reported that about 50% of his sample identified dysphagia as a symptom of GERD. Heartburn and regurgitation are the two most common symptoms of gastroesophageal reflux disease, which may account for the high number of responders who correctly identified them. Heartburn can be distinguished by a severe retrosternal burning sensation that lasts for many minutes. Patients may get angina-like chest discomfort as a result of reflux [26].

In our study, multiple factors were associated with knowledge regarding GERD. Higher age was associated with higher knowledge of GERD (p = 0.035). Knowledge was significantly lower among men than women (p = 0.007). Respondents with a high-school education had a significantly lower knowledge score than respondents with a university education (p = 0.063) although the association was statistically significant at the 0.1% level. The average knowledge score was significantly higher among students than respondents working as government employees (p = 0.003). In previous studies, the degree of disease knowledge differed significantly according to age (P < 0.001), education (P < 0.001), income (P = 0.028), and occupation (P < 0.001). In the multivariate analysis using multiple logistic regression, the higher knowledge score group tended to have higher education and a professional occupation [26]. Another study reported that the higher the level of education, the higher the score they got, and participants had gained most of their knowledge from the internet and books [27]. A Saudi study reported that gender and nationality both had a substantial impact on GERD awareness. Saudis had 70% awareness while non-Saudis had 25%; in addition, there was a substantial link between marital status, employment, and income level and the amount of GERD awareness [37]. A Turkish study showed a substantial number of unrecognized and untreated GERD patients, as well as a high incidence of GERD among economically disadvantaged groups due to a lack of knowledge, unhealthy lifestyle, and behaviors. This might be explained by the fact that low-income individuals do not have access to the communication tools that the contemporary world uses to raise awareness, such as televisions and cellphones [39].

## Conclusions

The study shows a relatively good knowledge level compared to previously reported figures in Saudi Arabia and worldwide. A GERD diagnosis is significantly associated with a high knowledge score about the disease and its associated factors. Educational programs for GERD should be increased in Saudi Arabia as more health conferences and teaching more school students about the disease should be highlighted to increase general knowledge about the disease in the KSA.
